# The Association of *Toxoplasma gondii* IgG and Liver Injury in US Adults

**DOI:** 10.3390/ijerph19127515

**Published:** 2022-06-19

**Authors:** Amani Babekir, Sayed Mostafa, Radiah C. Minor, Leonard L. Williams, Scott H. Harrison, Emmanuel Obeng-Gyasi

**Affiliations:** 1 Department of Built Environment, North Carolina A&T State University, Greensboro, NC 27411, USA; aebabekir@aggies.ncat.edu; 2 Environmental Health and Disease Laboratory, North Carolina A&T State University, Greensboro, NC 27411, USA; 3 Department of Mathematics and Statistics, North Carolina A&T State University, Greensboro, 27411 NC, USA; sabdelmegeed@ncat.edu; 4 Department of Animal Sciences, North Carolina A&T State University, Greensboro, NC 27411, USA; rcminor@ncat.edu; 5 Center for Excellence in Post-Harvest Technologies, North Carolina A&T State University, Kannapolis, NC 28081, USA; llw@ncat.edu; 6 Department of Biology, North Carolina A&T State University, Greensboro, NC 27411, USA; scotth@ncat.edu

**Keywords:** ALT, AST, ALP, CLD, NAFLD, liver disease, biomarker, *Toxoplasma*, *T. gondii*

## Abstract

Background: *Toxoplasma gondii* (*T. gondii*) is a ubiquitous obligatory intracellular parasite which infects over 40 million Americans and causes toxoplasmosis. Inside the human body, *T. gondii* can damage tissues and invade vital organs. Methods: This study evaluated the association of *T. gondii* infection and liver disease using data from the National Health and Nutrition Examination Survey (NHANES) 2009–2010, with a sample size of 3371 participants (age 20–80 years). *Toxoplasma* infection was determined by the level of *T. gondii* IgG antibody in serum samples. Liver disease was assessed by liver injury biomarkers and the Fatty Liver Index (US-FLI). The evaluation of the association between *T. gondii* infection and liver disease included the calculation of the Mantel–Haenszel risk ratio (RRMH), Rho-Scott chi-square bivariate analyses, design-based t-tests, and linear and logistic regression models which were adjusted for demographic and anthropometric covariates. Results: Mean levels of aspartate aminotransferase (AST) and alkaline phosphatase (ALP) were significantly more elevated in the *T. gondii* IgG-positive (IgG+) participants as compared to *T. gondii*-negative (IgG−) participants, *p* = 0.0435 and 0.0310, respectively. In linear regression analysis, exposure to *T. gondii* IgG+ had statistically significant positive associations with AST (*p* = 0.0211), alanine aminotransferase (ALT) (*p* = 0.0221), and gamma-glutamyl transferase (GGT) (*p* = 0.0258) after adjusting for BMI, age, gender, and race. *T. gondii* exposure was associated with an elevated relative risk of chronic liver disease (CLD) (RRMH = 1.26, 95% CI: 1.05–1.51). This association was more pronounced in certain occupations, such as construction, agriculture, forestry, and fishing, where *Toxoplasma* infection is more common (*p* = 0.0477). Moreover, *Toxoplasma* infection increased the odds of nonalcoholic fatty liver disease (NAFLD) (OR = 6.99, 95% CI = 1.85–26.32, *p* = 0.0237). Conclusion: *T. gondii* IgG+ antibody was significantly associated with liver injury biomarkers (ALT, AST, GGT, and ALP) and an increased risk of CLD and NAFLD. Moreover, the association of *Toxoplasma* with CLD was more evident in specific occupations where the prevalence of *Toxoplasma* was high. The findings of this study provide insight into utilizing liver biomarkers and US-FLI to assess the health complications of *Toxoplasma* when imaging tests are not accessible.

## 1. Introduction

*Toxoplasma gondii (T. gondii*) is a parasite that infects a third of the world’s population, including 40 million Americans. The infection causes toxoplasmosis and is considered by the Centers for Disease Control and Prevention as the leading cause of death related to foodborne illnesses in the United States [1]. Although toxoplasmosis is asymptomatic in the majority of cases, chronic infections may cause damage to vital organs such as the heart, kidneys, and liver [2]. Furthermore, *T. gondii* strains have different genotypes that vary in virulence and geographical location, such as type I, II, and III, the most prevalent strains in the U.S. [3,4].

Wild and domestic felines are the definitive hosts for *T. gondii*, while warm-blooded animals, including humans, are intermediate hosts [5,6]. The lifecycle of this parasite includes three infectious forms: tachyzoites, bradyzoites (cyst), and sporozoites (oocyst) [5]. Infected felines excrete oocysts in their feces, contaminating the surrounding environment and infecting the intermediate hosts. Inside the intermediate host, *T. gondii* infects body tissues and forms cysts [7]. Humans contract *T. gondii* by eating raw meat containing *T. gondii* cysts or ingesting food contaminated with *T. gondii* oocyst, such as vegetables and milk [5,8,9]. The less frequent routes are organ transplants and congenital infections [9,10,11,12].

Several factors play a role in the susceptibility of humans to *T. gondii* infections and its health complications, including age, demographic characteristics, and immune system strength [9,11,13]. *T. gondii* infection is more prevalent in the United States (U.S.) among older people, Mexican Americans, other-Hispanics, and people who work in occupations such as mining, fishing, and construction [14,15].

*T. gondii* infection is clinically diagnosed by testing the level of *T. gondii* IgG and IgM antibodies, where *T. gondii* IgM indicates acute infection and the IgG antibodies indicate acute and latent infection [16,17,18]. The health complications of toxoplasmosis involve processes such as oxidative stress and inflammation [19,20,21]. In addition, *T. gondii* IgG antibody is associated with adverse levels of biomarkers indicative of disease, such as gamma-glutamyl transferase (GGT), C-reactive protein (CRP), triglycerides (TG), and markers of persistent albuminuria [15,22]. Toxoplasmosis is associated with several diseases, such as schizophrenia, cardiovascular diseases, obsessive-compulsive disorder, and chronic kidney disease [16,21,23,24,25].

In 2018, 4.5 million Americans were diagnosed with liver disease (1.8%), with 51,642 individuals dying of the illness in 2020 [26]. There are two types of liver disease, acute and chronic. Chronic liver disease (CLD) is a progressive deterioration of liver function [27]. Asymptomatic in the early stage, CLD can lead to irreversible damage to the liver and promote liver failure by limiting the liver’s ability to break down nutrients and filter toxic agents [28,29]. The accumulation of toxic agents and imbalance of lipoprotein and albumin may impair the immune system and expose the body to opportunistic parasites [30]. Liver diseases are further classified into three categories: alcohol-associated liver disease, non-alcoholic fatty liver disease (NAFLD), and viral hepatitis (hepatitis A, B, C, D, E). Alcohol-associated liver disease is damage of the liver due to prolonged alcohol consumption, while NAFLD is liver damage not associated with alcohol consumption [31]. NAFLD is the most common CLD in the U.S. [32]. While the major cause of NAFLD has not been clearly defined, several risk factors are identified, including high cholesterol, high TG, diabetes, and obesity [31,33].

The most common biomarkers used to evaluate liver injury are alanine aminotransferase (ALT), aspartate aminotransferase (AST), alkaline phosphatase (ALP), glutamyl transpeptidase (GGT), total bilirubin (TBIL), and lactate dehydrogenase (LDH). The levels of TBIL may correlate with the overall liver dysfunction, while ALP level is an indication of biliary damage, and high AST/ALT concentrations may indicate hepatocyte necrosis [34]. The NHANES survey monitors the trend of liver health among the U.S. population by testing survey participants using these biomarkers [35].

NAFLD is usually diagnosed with findings of hepatic steatosis through ultrasound, but without ultrasound hepatic steatosis could be predicted with the Fatty Liver Index (US-FLI), which has been validated using NHANES data [36]. The US-FLI incorporates various factors such as waist circumference, body mass index (BMI), GGT, and triglycerides to create an overall score. Thus, an overall score higher than 59 is considered predictive of NAFLD when the presence of other causative agents (alcohol drinking, chronic hepatitis B or C, and history of liver disease) are absent [37]. This US-FLI revealed that the prevalence of NAFLD in the U.S. population has increased from 18% to 31% within two decades (1988–2012) [36].

CLD is a gradual process of hepatic fibrosis, which involves the formulation of extracellular matrix triggered by liver injury [27]. The hepatic fibrosis process is initiated by hepatic stellate cells that induce the inflammatory receptors, including chemokine receptors and ICAM-1. Consequently, the hepatic cells respond to these inflammatory cytokines by accumulating extracellular matrix [38].

Inside the human body, *T. gondii* is capable of invading liver tissues and causing cell injury and inflammation. Furthermore, *Toxoplasma* infection is associated with several liver pathological conditions, such as necrosis, hepatomegaly, granuloma, and hepatitis [23,39,40]. Additionally, chronic liver disease may weaken the immune system and increase the susceptibility to *Toxoplasma* infection [24,41]. A case-control study (75 patients vs. 150 control) in a Northern Mexican population found no correlation between liver disease and *Toxoplasma* [42]. However, infection with *T. gondii* was linked with a higher prevalence of NAFLD, which was diagnosed with liver ultrasonography, cirrhosis, and CLD [43,44,45].

### Purpose and Hypothesis

Few studies have examined the association between *Toxoplasma* and the risk of liver disease in the U.S. [43] population, and very little research has explored this association with biomarkers linked to liver injury [24,43]. Consequently, the objective of this study was to thoroughly examine this association using the most common liver injury biomarkers and the liver function index based on a representative sample of the U.S. population. This study hypothesized that *Toxoplasma* infection is associated with liver injury and disease and that the type of occupation may contribute to the level of this association. The objectives of this study were to examine:

1. The association between *T. gondii* IgG and liver biomarkers (AST, ALT, ALP, TBIL, GGT, and LDH).
2. The association between *T. gondii* IgG and CLD.
3. The assessment of the role of occupation in the association between *T. gondii* IgG and CLD.
4. The association between *T. gondii* IgG and NAFLD.

## 2. Materials and Methods

### 2.1. Study Population

The study sample was obtained from NHANES 2009–2010 participants with an inclusion criterion of individuals (age 20–80 years) who did not consume five or more alcoholic drinks every day [46,47]. The sampled participants were tested for *T. gondii* and liver injury biomarkers. The NHANES 2009–2010 participants were selected through a multistage stratified design to represent the non-institutionalized U.S. population. The protocols of NHANES 2009–2010 were approved by the National Center for Health Statistics of the Centers for Disease Control and Prevention Institutional Review Board [48].

### 2.2. Blood and Serum Collection, T. gondii IgG Evaluation

Surplus serum samples collected in the NHANES 2009–2010 for sampled persons were tested for *T. gondii* antibodies at the CDC’s Parasitic Diseases Serology Laboratory. *T. gondii* IgG antibody was tested (IU/mL) with the *Toxoplasma* IgG enzyme immunoassay kit (Bio-Rad, Redmond, VA) following the manufacturer’s protocol. Measurements ≥ 33 IU/mL were considered as positive *T. gondii* (IgG+) and < 27 IU/mL were considered negative (IgG−). Measurements ≥ 27 and <33 IU/mL were confirmed as negative after double testing [35].

### 2.3. Liver Injury Biomarkers

Samples were collected by specialists during the NHANE survey and stored following the NHANE 2009–2019 Laboratory Procedure Manual. The collected serum samples were shipped to the Collaborative Laboratory Services for analysis, where a DxC800 chemistry analyzer was used to measure liver injury biomarkers as a part of the routine biochemistry profile. The DxC800 used an enzymatic, kinetic rate, or timed-endpoint method to measure AST, ALT, ALP, TBIL, GGT, LDH, and TG [35].

### 2.4. Chronic Liver Disease (CLD)

CLD was classified in this study as those that meet the criteria/do not meet the criteria for CLD based on gender and the level of ALT or AST [49]. Positive CLD was defined as:

- Men: ALT > 40 U/L or AST > 37 U/L
- Women: ALT or AST > 31 U/L

### 2.5. US-FLI and NAFLD

US-FLI was used to predict NAFLD because abdominal ultrasound results were unavailable in the data used. The US-FLI has been validated as a predictor of NAFLD in the U.S. [47]. The US-FLI is calculated using a logistic regression model for the probability of having NAFLD as a function of BMI, waist circumference, GGT, and triglycerides (TG), as shown in the equation below [36]. In our study, participants with a US-FLI score greater than 59 were predicted to have NAFLD [36]. Participants with positive hepatitis A, B, C, or D were excluded (1878 participants).

$$\mathrm{FLI}\;=\frac{\exp\left(A\right)}{1+\exp\left(A\right)}\times 100,$$

where,

A =0.953∗log(TG)+0.139∗BMI+0.718∗log(GGT)+0.053∗waist circumference−15.745

and log(·) is the natural logarithm.

### 2.6. Covariates

Additional variables considered in the analysis were gender, age, race/ethnicity, and occupation (longest job). The occupation variable had 22 levels (types of occupations) which we grouped into two groups: group 1 included the occupations which were found to be associated with *Toxoplasma* infection in previous studies [14,22,50] and group 2 included all other types of occupations. Specifically,

- Group 1: Agriculture, forestry, fishing, mining, construction.
- Group 2: Other.

### 2.7. Statistical Analysis

The *Survey* package in R (version 4.0.2; R Foundation for Statistical Computing, Vienna, Austria) was used in the analyses considering the sampling design and survey weights. Log transformation was used to overcome the skewed distribution of continuous variables, including *T. gondii* IgG antibody, AST, ALT, ALP, TBIL, GGT, and LDH. The analysis methods included Mantel–Haenszel risk ratio estimates [51], Rho-Scott chi-square bivariate analyses, design-based *t*-tests, and linear and logistic regressions. A *p*-value below 0.05 was considered statistically significant in all the analyses.

## 3. Results

### 3.1. Characteristics of Study Participants

The study used a sample of 3371 NHANES participants, which included participants (age 20–80 years) who have been tested for *T. gondii* and liver injury biomarkers. Table 1 provides summary statistics (survey-weighted percentages for categorical variables and survey-weighted means, and their corresponding standard errors, for quantitative variables) for the different variables used in the analysis. The sample consisted of 47.2% males and 52.8% females, with 14.3% of sample participants classified as *T. gondii*-positive (IgG+). The mean age of participants was 46.9 years, and 10.6% of participants worked in agriculture, forestry, fishing, mining, or in the construction occupation. The sample included Mexican Americans (7.7%), other Hispanics (5.0%), non-Hispanic whites (72.8%), non-Hispanic blacks (9.1%), and the other race category (5.4%).

### 3.2. Association of Toxoplasma and Liver Injury Biomarkers

**Figure 1:**
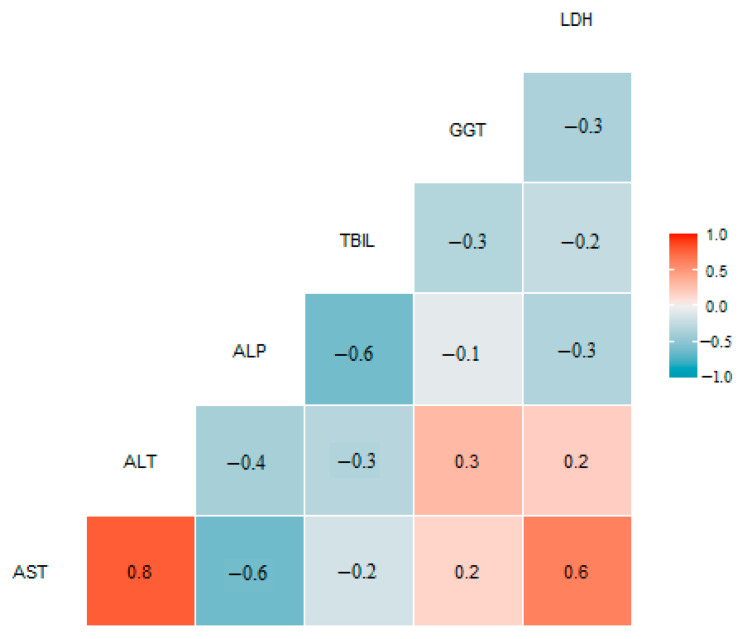
Correlation matrix of liver biomarkers (Pearson correlation coefficient).

The correlation of the liver injury biomarkers was investigated with a correlation matrix (Figure 1). A correlation existed between AST and LDH (Pearson correlation coefficient $\hat{\rho }$ = 0.6), and the highest correlation was apparent between ALT and AST (Pearson correlation coefficient $\hat{\rho }$ = 0.8).

The comparison of the liver injury biomarkers between *T. gondii* IgG+ and IgG− participants was conducted using the design-based *t*-test (Table 2). The AST and ALP averages were significantly higher in the *T. gondii* IgG+ participants compared to the IgG− participants, *p* = 0.0435 and 0.0310, respectively, while the TBIL mean was significantly lower in the *T. gondii* IgG+ participants than the IgG− participants (*p* = 0.0032). The data did not show evidence of significant differences in the remaining biomarkers by *T. gondii* exposure status.

Multiple linear regression models were fit to assess the association between *T. gondii* IgG antibody status and each liver biomarker after adjusting for demographic variables and BMI (Table 3). Two models were created for each biomarker. The first model adjusted for BMI only, while the second model adjusted for BMI, age, gender, and race. *T. gondii* IgG+ had a significant positive association with AST (*p* = 0.0211), ALT (*p* = 0.0221), and GGT (*p* = 0.0258). In contrast, *T. gondii* IgG+ had a significant negative correlation with TBIL (*p* = 0.0103). No significant correlation was found between *T. gondii* IgG antibody status and ALP or LDH.

### 3.3. Association of Toxoplasma and CLD Status

The percentage of sample participants meeting the criteria for CLD was 16.6%. The relative risk of CLD by *T. gondii* exposure is detailed in Table 4. The age-adjusted risk ratio was calculated with the Mantel–Haenszel formula [52]. The overall risk ratio did not indicate a significant association between CLD and *T. gondii* exposure; however, the age-adjusted relative risk suggested that the *T. gondii* exposure significantly increased the risk of CLD (RR_MH_ = 1.26, 95% CI: 1.05–1.51).

To consider other possible variables in addition to age, we used regression models. Table 5 and Table 6 show binomial logistic regression models developed to study the association between *T. gondii* exposure and CLD. The first model (Table 5) adjusts for age, gender, race/ethnicity, and BMI. This model did not result in a statistically significant association between *T. gondii* exposure and CLD; however, it indicated a strong association between CLD and demographic factors, especially gender (*p* ≤ 0.0001), which was factored into the calculation of CLD. The second model (Table 6) excluded the gender and BMI factors which had the strongest association with CLD. The association of *Toxoplasma* was significant in this model (OR = 2.80, 95% CI = 1.30– 6.03, *p* = 0.0271).

### 3.4. Occupation Factor

**Figure 2:**
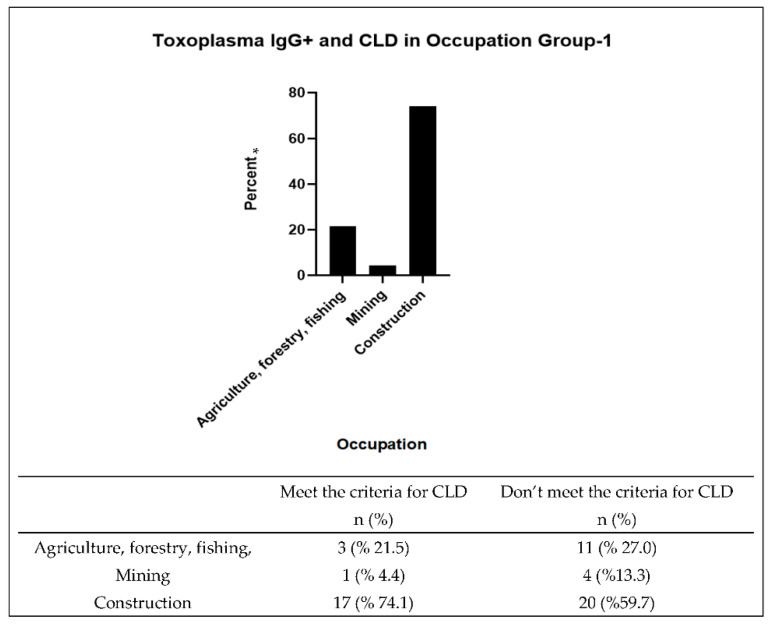
Occupation Group 1 *Toxoplasma* and CLD. * Percent of T. gondii IgG+ with CLD.

Multiple models were built to investigate the effect of the type of occupation on the association of *Toxoplasma* and liver injury biomarkers and CLD. Table 7 shows a binomial logistic regression model adjusted for occupation and BMI. The model did not demonstrate an association of *Toxoplasma* and CLD; however, it showed a strong association between occupation type and CLD (*p* = 0.0001). Those in occupations with a low occupational exposure risk to *Toxoplasma* had 52% lower odds of CLD than those in high-risk occupations (OR = 0.48, 95% CI = 0.29–0.78); however, this model did not show an interaction between occupation and *Toxoplasma*. The portion of *T. gondii* IgG+ among those meeting the criteria for CLD was higher among Group 1 compared to Group 2 (33.3% vs. 13.1%, Table 8). In addition, those meeting the criteria for CLD were significantly more prevalent in Group 1 than Group 2 (*p* ≤ 0.0001), and Group 2 had a significantly higher number of *T. gondii* IgG+ participants (*p* = 0.0063), while in Group 2 the number of *T. gondii* IgG+ participants was not significantly high when compared to *T. gondii* IgG+ participants in the sample group. Therefore, two binomial logistic regression models were created for each group of occupation (Table 9). The model of Group 1 revealed that the *T. gondii* IgG antibody level had a significant positive correlation with CLD (*p* = 0.0477). On the other hand, the model of Group 2 did not show the association of *T. gondii* IgG antibody with CLD. In Group 1, the occupation with the highest percentage of *T. gondii* IgG+ and those that meet the criteria for CLD was the construction occupation (Figure 2).

### 3.5. Association of Toxoplasma and US-FLI

A further analysis was conducted using US-FLI which excluded participants positive for hepatitis A, B, C, or D. Table 10 shows that the proportion of FLI ≥ 60 was significantly higher among *T. gondii* IgG+ participants when compared with *T. gondii* IgG− participants (57.5% vs. 44.1%, *p* = 0.0064).

In addition, a binomial logistic regression model was created to investigate the association of *Toxoplasma* and NAFLD in the absence of viral hepatic liver diseases and adjusted for BMI and demographic factors (Table 11). The model showed that exposure to *Toxoplasma* increased the odds of positive NAFLD (OR = 6.99, 95% CI = 1.85–26.32, *p* = 0.0237) and the interaction of age in this association (*p* = 0.0210). Moreover, age, gender, race, and BMI were associated with NAFLD.

## 4. Discussion

### 4.1. Overview of Results and Implications

Previous studies investigated the association of *Toxoplasma* and liver disease in animals and global populations. Nevertheless, the associated risk of *Toxoplasma* infection and liver dysfunction in the U.S. population was not thoroughly investigated.

A previous study which utilized a sample of the U.S. population from NHANES III data (1988–1994) indicated no significant difference in the level of liver injury biomarkers (AST and ALT) between *T. gondii* IgG+ and IgG− groups, but ALP and GGT were significantly different [43]. Our analysis investigated this association using more recent data (2009–2010), additional analysis, and inclusion of critical covariates. Our analysis revealed that the AST and ALP averages were significantly higher in the *T. gondii* IgG+ participants compared to the *T. gondii* IgG− participants, *p* = 0.0435 and 0.0310, respectively. Serum samples collected from patients linked to a toxoplasmosis outbreak in Wisconsin showed that 86% (6 out of 7) of the patients had either elevated AST or ALT [52]. The data presented here supported this finding and revealed the strong correlation between AST and ALT. Furthermore, a positive association between *Toxoplasma* and AST (*p* = 0.0211), ALT (*p* = 0.0221), and GGT (*p* = 0.0258) was revealed after adjusting for BMI, age, gender, and race. Prior studies linked *T. gondii* IgG+ and adverse levels of several clinical biomarkers related to heart and kidney function, including blood pressure, TG, low-density lipoprotein cholesterol, high-density lipoprotein cholesterol, albuminuria, and GGT [15,21,22].

The *Toxoplasma* association was further investigated in this study using the elevated level of AST or ALT in the form of meet the criteria/do not meet the criteria for CLD.

The age-adjusted relative risk indicated that the *Toxoplasma* infection increased the risk of CLD (RR_MH_ = 1.26, 95% CI: 1.05–1.51). In addition, the binomial logistic regression model adjusted for age and race/ethnicity showed that the *T. gondii* IgG+ group had a higher odds ratio of CLD than the negative group (OR = 2.80, 95% CI = 1.30–6.03, *p* = 0.0271). This finding matches the work of El-Sayed and colleagues, who conducted a case-control study in Egypt and found that patients with CLD had higher prevalence of *T. gondii* infection [24].

Previous studies showed the prevalence of *T. gondii* in specific occupations including agriculture, fishing, mining, and construction (Group 1) [14,22,50]. Therefore, the association of *Toxoplasma* and CLD and how it related to occupation was investigated in this study. The results showed that Group 1 had a significantly higher prevalence of those meeting the criteria for CLD and *Toxoplasma* infection than Group 2. In addition, participants who worked in occupations other than those in Group 1 had a lower CLD risk than participants in Group 1 (OR = 0.48, 95% CI = 0.29–0.78, *p* = 0.0133). Moreover, the exposure to *Toxoplasma* infection was associated with those meeting the criteria for CLD in Group 1 (*p* = 0.0477), especially among construction workers (74.1%). This finding could be explained by earlier studies that associated Group 1 with exposure to environmental contamination with *T. gondii* oocysts and a high prevalence of parasitic infections and chronic diseases among low-income workers [14,47,50].

A further investigation of the association between *Toxoplasma* and liver dysfunction was conducted in this study using US-FLI. The results showed that *T. gondii* IgG+ participants had significantly higher US-FLI than the *T. gondii* IgG− participants and had increased odds of having NAFLD even after adjusting for the other risk factors, such as age, gender, race, and BMI, and without the presence of heavy alcohol drinking and viral hepatitis (OR = 6.99, 95% CI = 1.85–26.32, *p* = 0.0237).

The association of *Toxoplasma* and liver dysfunction confirmed in this study could have two directions: the infection with *Toxoplasma* increases the risk of liver injury or liver dysfunction increases the risk of *T. gondii* infection. Previous studies linked *Toxoplasma* infection to liver injury and its association with necrosis, hepatomegaly, granuloma, and hepatitis [23,39,40]. On the other hand, CLD increases the susceptibility to *Toxoplasma* infection [43,44,45].

*Toxoplasma* infection was previously associated with several diseases in the U.S. population, including obsessive-compulsive disorder, schizophrenia, cardiovascular disease, and chronic kidney disease, and our study showed that *Toxoplasma* infection was associated with liver disease as well when adjusting for critical covariates.

In addition, the findings of this study provide insight into utilizing liver biomarkers and US-FLI to assess the health complications of *Toxoplasma* when imaging tests are not accessible.

This study used greater than 5 drinks/day to rule out AFLD in CLD, excluding the heavy drinking cases and maintaining the balance of the data [46,47]. However, future studies may investigate this association using a lower threshold [53].

### 4.2. Implications of Findings

*Toxoplasma* is considered as one of the neglected parasites in the U.S. This study contributes to bridging the gap in the literature on the implications of parasite infection on liver injury as assessed by liver injury biomarkers and the US-FLI.

### 4.3. Limitation

Although this study showed an association between *Toxoplasma* infection and liver dysfunction, additional research is needed to study the direction of this association. Considering the limitation of the cross-sectional design of the study, a longitudinal study will provide a better understanding of the health complications of *Toxoplasma* infection and the direction of the association. Moreover, a larger sample size, lower drinking threshold, and inclusion of additional factors may provide a better explanation for this association.

## 5. Conclusions

*T. gondii* IgG+ was significantly associated with an adverse level of liver injury biomarkers (ALT, AST, GGT, and ALP), an increased risk of CLD, and NAFLD. Moreover, the association of *Toxoplasma* with CLD was more evident in specific occupations where the prevalence of *Toxoplasma* was high.

## Figures and Tables

**Table 1:** Summary statistics for the characteristics of sample participants, *n* = 3371.

Variables	Description	ALL		*T. gondii* IgG−		*T. gondii* IgG+	
		*n*	Weighted Percentage/Mean (SE)	*n*	Weighted Percentage/Mean (SE)	*n*	Weighted Percentage/Mean (SE)
* T. gondii * IgG antibody	<33 IU/mL (IgG−)	2761	85.7	2761	85.7	0	
	≥33 IU/mL (IgG+)	610	14.3	0		610	14.3
	Average	3371	16.4 (1.05)				
Gender	Male	1626	47.2	1285	45.8	341	55.3
	Female	1745	52.8	1476	54.2	269	44.7
Age		3371	46.9 (0.56)	2761	45.9 (0.56)	610	52.8 (0.92)
Race/ethnicity	Mexican American	575	7.7	463	7.2	112	10.3
	Other Hispanic	353	5.0	235	4.1	118	11.1
	Non-Hispanic White	1783	72.8	1524	74.5	259	62.7
	Non-Hispanic Black	509	9.1	409	8.7	100	11.2
	Other Race	151	5.4	130	5.5	21	4.7
BMI		3371	28.8 (0.15)	2761	28.6 (0.13)	610	29.5 (0.33)
Occupation	Group 1	204	10.6				
	Group 2	1657	89.4				
Liver Injury Biomarkers							
AST		3371	25.7 (0.21)				
ALT		3371	25.5 (0.34)				
ALP		3371	66.0 (0.35)				
TBIL		3371	0.77 (0.01)				
GGT		3371	25.2 (0.65)				
LDH		3371	130.4 (1.01)				
CLD	Negative	2830	83.4				
	Positive	541	16.6				

**Table 2:** Means (standard error) of liver injury biomarkers by *T. gondii* exposure status (IgG+/IgG−).

Variable	*T. gondii* IgG−	*T. gondii* IgG+	*p*-Value
	Mean (SE)	Mean (SE)	
AST	25.5 (0.20)	27.1 (0.75)	0.0435
ALT	25.3 (0.37)	26.3 (0.45)	0.0668
ALP	65.7 (0.39)	67.7 (0.75)	0.0310
TBIL	0.8 (0.00)	0.7 (0.00)	0.0032
GGT	25.1 (0.70)	26.1 (0.79)	0.2527
LDH	129.9 (0.93)	133.8 (2.73)	0.1338

**Table 3:** Results of linear regression models for liver biomarkers and *T. gondii* IgG antibody—adjusted for demographic and anthropometric factors.

Response	Covariates	*T. gondii* IgG (Ref. = IgG−)	
		Coefficient (SE)	*p*-Value
AST	BMI	0.01 (0.00)	0.0009
	Age, gender, race, BMI	0.03 (0.01)	0.0211
ALT	BMI	0.00 (0.00)	0.2220
	Age, gender, race, BMI	0.01 (0.01)	0.0221
ALP	BMI	0.01 (0.01)	0.1930
	Age, gender, race, BMI	0.01 (0.01)	0.3356
TBIL	BMI	−0.01 (0.00)	0.0103
	Age, gender, race, BMI	−0.01 (0.00)	0.0992
GGT	BMI	0.01 (0.01)	0.0925
	Age, gender, race, BMI	0.04 (0.02)	0.0258
LDH	BMI	0.01 (0.00)	0.1720
	Age, gender, race, BMI	0.02 (0.01)	0.1494

**Table 4:** Relative risk of CLD by *T. gondii* exposure status (IgG+/IgG−).

*Toxoplasma* IgG Antibody	CLD	
	Meet the Criteria for CLD n (Weighted %)	Do not Meet the Criteria for CLD n (Weighted %)
IgG−	430 (84.1)	2331 (86.1)
IgG+	111 (15.9)	499 (13.9)
Risk ratio (R_crude_) = 1.16 (95% CI: 0.97–1.40)		
Age-adjusted risk ratio (R_RMH_) = 1.26 (95% CI: 1.05–1.51)		

**Table 5:** Results of binomial logistic regression for CLD and *T. gondii* (IgG+/IgG−)—adjusted for BMI and demographic factors.

	CLD			
Sample Size = 3357	Odds Ratio	OR 95% Confidence Interval		*p*-Value
*T. gondii* IgG+ (Ref. = IgG−)	1.88	0.57	6.22	0.3354
Age	0.99	0.98	0.99	0.0239
Gender (Ref. = Male)				
Female	0.00	0.00	0.00	<0.0001
Race/ethnicity (Ref. = Mexican American)				
Other Hispanic	0.68	0.42	1.11	0.1679
Non-Hispanic White	0.72	0.54	0.97	0.0669
Non-Hispanic Black	0.74	0.52	1.05	0.1401
Other Race	0.64	0.30	1.01	0.2960
BMI	1.05	1.03	1.07	0.0005
*T. gondii* IgG+ x Age	0.99	0.52	1.54	0.1401
Constant	0.01	0.19	0.52	0.0029
AIC = 2096				
Pseudo R-Square = 0.26				

**Table 6:** Results of binomial logistic regression for CLD on *T. gondii* (IgG+/IgG−)—adjusted for the demographic factors (excluding gender and BMI).

	CLD			
Sample Size = 3371	Odds Ratio	OR 95% Confidence Interval		*p*-Value
*T. gondii* IgG+ (Ref. = IgG−)	2.80	1.30	6.03	0.0271
Age	0.99	0.98	0.99	0.0093
Race/ethnicity (Ref. = Mexican American)				
Other Hispanic	0.65	0.41	1.00	0.0851
Non-Hispanic White	0.65	0.49	0.84	0.0101
Non-Hispanic Black	0.64	0.46	0.89	0.0265
Other Race	0.60	0.30	1.23	0.2014
*T. gondii* IgG+ x Age	0.98	0.97	0.99	0.0230
Constant	0.47	0.35	0.63	0.0007
AIC = 3141				
Pseudo R-Square = 0.01				

**Table 7:** Results of binomial logistic regression for CLD on *T. gondii* (IgG+/IgG−)—adjusted for BMI, age, and occupation (Group 1/Group 2).

	CLD			
Sample Size = 1851	Odds Ratio	OR 95% Confidence Interval		*p*-Value
*T. gondii* IgG+	2.23	1.01	4.91	0.0715
Occupation				
Group 1 (ref)				
Group 2	0.48	0.29	0.78	0.0133
BMI	1.02	1.01	1.04	0.0607
Age	0.98	0.97	0.99	0.0013
*T. gondii* IgG+ x Occupation	0.40	0.13	1.22	0.1378
Constant	0.35	0.09	0.41	0.0008
AIC = 1349				
Pseudo R-Square = 0.02				

**Table 8:** *T. gondii* (IgG+/IgG−) vs. CLD per occupation (Group 1/Group 2).

	Occupation (Group 1)		Occupation (Group 2)	
	CLD *		CLD	
*Toxoplasma* IgG Antibody **	Meet the criteria for CLD n (Weighted %)	Do not meet the criteria for CLD n (Weighted %)	Meet the criteria for CLD n (Weighted %)	Do not meet the criteria for CLD n (Weighted %)
IgG+	21 (33.3)	35 (19.5)	31 (13.1)	270 (15.9)
IgG−	36 (66.7)	112 (80.5)	170 (86.9)	1186 (84.1)

* Significant: those that meet the criteria for CLD in Group 1 compared to Group 2 (*p* ≤ 0.0001). ** Group 2 had a significantly higher number of *T. gondii* IgG+ participants (*p* = 0.0063), while in Group 2 the number of *T. gondii* IgG+ participants was not significantly high when compared to *T. gondii* IgG+ participants in the sample group.

**Table 9:** Binomial logistic regression model for CLD on *T. gondii* IgG antibody—occupation (Group 1/Group 2) *.

	Occupation (Group 1)		Occupation (Group 2)	
	CLD		CLD	
	Coefficient (SE)	*p*-Value	Coefficient (SE)	*p*-Value
*Toxoplasma* IgG antibody	0.67 (0.34)	0.0477	−0.08 (0.21)	0.6882
Age	−0.01 (0.00)	0.0801	−0.02 (0.00)	0.0001
BMI	0.01 (0.00)	0.6973	0.015 (0.00)	0.1158
Constant	−0.67 (0.94)	0.4752	−1.64 (0.33)	<0.001
Sample size	203		1648	
AIC	242.6		1208.3	
Pseudo R-Square	0.03		0.01	

* The survey design was not utilized in this analysis due to the limited number of stratums in the groups.

**Table 10:** US-FLI per *T. gondii* (IgG+/IgG−).

*Toxoplasma* IgG Antibody	US-FLI	
	FLI < 60 (%)	FLI ≥ 60 (%)
IgG−	55.9	44.1
IgG+	42.5	57.5
*p*-value = 0.0064		

Sample size = 1878, participants positive for hepatitis A, B, C, or D were excluded.

**Table 11:** Results of binomial logistic regression for NAFLD on *T. gondii* (IgG+/IgG−)—adjusted for BMI and demographic factors.

	NAFLD			
Sample Size = 1845	Odds Ratio	OR 95% Confidence Interval		*p*-Value
* T. gondii * IgG+	6.99	1.85	26.32	0.0237
Age	1.03	1.02	1.04	0.0007
Gender				
Male (ref)				
Female	0.21	0.14	0.31	0.0001
Race/ethnicity				
Mexican American (ref)				
Other Hispanic	0.39	0.10	1.46	0.2079
Non-Hispanic White	0.45	0.19	1.04	0.1071
Non-Hispanic Black	0.16	0.06	0.42	0.0079
Other Race	1.15	0.23	5.67	0.8631
BMI	2.03	1.92	2.15	<0.0001
* T. gondii * IgG+ x Age	0.97	0.95	0.98	0.0201
Constant	0.00	0.00	0.00	0.000
AIC = 891.7				
Pseudo R-Square = 0.58				

## Data Availability

The NHANES dataset is publicly available online, accessible at the CDC website [54].
